# HerbKG: Constructing a Herbal-Molecular Medicine Knowledge Graph Using a Two-Stage Framework Based on Deep Transfer Learning

**DOI:** 10.3389/fgene.2022.799349

**Published:** 2022-04-27

**Authors:** Xian Zhu, Yueming Gu, Zhifeng Xiao

**Affiliations:** ^1^ School of Information Management, Nanjing University, Nanjing, China; ^2^ School of Health Economics and Management, Nanjing University of Chinese Medicine, Nanjing, China; ^3^ School of Computing and Information Systems, Faculty of Engineering and Information Technology, University of Melbourne, Parkville, VIC, Australia; ^4^ School of Engineering, Penn State Erie, The Behrend College, Erie, PA, United States

**Keywords:** biobert, knowledge graph, herb, chemical, disease, gene, BERT, ontology

## Abstract

Recent advances have witnessed a growth of herbalism studies adopting a modern scientific approach in molecular medicine, offering valuable domain knowledge that can potentially boost the development of herbalism with evidence-supported efficacy and safety. However, these domain-specific scientific findings have not been systematically organized, affecting the efficiency of knowledge discovery and usage. Existing knowledge graphs in herbalism mainly focus on diagnosis and treatment with an absence of knowledge connection with molecular medicine. To fill this gap, we present HerbKG, a knowledge graph that bridges herbal and molecular medicine. The core bio-entities of HerbKG include herbs, chemicals extracted from the herbs, genes that are affected by the chemicals, and diseases treated by herbs due to the functions of genes. We have developed a learning framework to automate the process of HerbKG construction. The resulting HerbKG, after analyzing over 500K PubMed abstracts, is populated with 53K relations, providing extensive herbal-molecular domain knowledge in support of downstream applications. The code and an interactive tool are available at https://github.com/FeiYee/HerbKG.

## 1 Introduction

A knowledge graph (KG) serves as a useful tool to represent real-world semantic phenomena in an organized way (Wang et al., 2017). Specifically, a KG consists of a collection of three tuples, each of which follows a format of [head, tail, relation]. The head and tail specify an entity pair, and the relation defines how the two entities are semantically related. Both entities and relations can have domain-specific properties. A broad spectrum of large-scale KGs encoding generic knowledge, such as YAGO3 (Mahdisoltani et al., 2014), Freebase (Bollacker et al., 2008), DBpedia (Auer et al., 2007), and BabelNet (Navigli and Ponzetto, 2012), have been developed and have created massive value that benefits a variety of downstream applications, such as knowledge visualization (Kerdjoudj and Curé, 2015) and reasoning (Chen et al., 2020), information retrieval (Wise et al., 2020), and question answering (Saha et al., 2018). Furthermore, domain-specific KGs have also gained extensive interests (Ernst et al., 2014) by domain experts who desire to have efficient access to high-quality domain knowledge. For instance, a biomedical KG allows researchers and medical practitioners to mine and discover complex interactions between millions of bio-entities (e.g., chemicals, genes, and diseases), facilitating academic knowledge query and clinical decision making (Su et al., 2021; Zheng et al., 2021). Adding such domain knowledge into existing healthcare applications can greatly improve the quality and efficiency of current medical operations and IT systems.

As a sub-field of medicine, herbal medicine, also referred to as herbalism, is the study of pharmacognosy and the use of medicinal plants, forming the basis of traditional medicine that has been existing and evolving for over five thousand years across multiple continents and countries, including Africa, Americas, ancient Egypt, Greece, China, and India (Wikipedia contributors, 2004c). In the modern era, herbalism has received criticism and skepticism due to the lack of strong evidence of efficacy and safety found in high-quality scientific publications. Currently, herbalism is still the primary health care in many underdeveloped regions and is widely used to treat chronic diseases, such as diabetes (Egede et al., 2002), cancer (Burstein et al., 1999), end-stage kidney disease (Roozbeh et al., 2013), and asthma (Szelenyi and Brune, 2002). In the past decades, more and more researchers have taken a modern scientific approach to investigating the biological function of herbs and herbal contents, validating the interconnection between herbs, extracted chemicals, diseases, and genes (Babu et al., 2007; Brackman et al., 2008). These studies bridge modern molecular medicine and traditional herbalism, which provides more evidence to support the medicinal usage of herbs, opening a promising direction to boost the development of herbalism in the 21st century. In addition, these studies provide valuable domain knowledge in herbalism that should be restructured for building a herb KG.

Existing efforts of herb KG construction mainly focus on the diagnosis and treatment side of herbalism. Wang et al. (Wang et al., 2019) propose a Knowledge Graph Embedding Enhanced Topic Model (KGETM) for traditional Chinese medicine (TCM). The used KG in the study considers relations between symptoms, syndromes, treatments, and herbs to support a herb recommendation application. Zheng et al. (Zheng et al., 2020) have developed a TCM KG that stores herbs, therapies, prescriptions, diseases, syndromes, symptoms, and the relations between them. Another group (Somé et al., 2019) has developed the West African Herbal-based Traditional Medicine KG consisting of 143 identified West African medicinal plants and 108 recipes to treat 110 diseases and symptoms. Similar herb KGs that aim to facilitate prescription can be found in literature (Liu et al., 2018; Miao et al., 2018; Gong et al., 2021). On the other hand, prior efforts have also investigated generic biomedical KGs. Zheng et al. (Zheng et al., 2021) propose PharmKG, which consists of 500,000 relations between genes, drugs, and diseases. Another study by Su et al. (Su et al., 2021) proposes the Cornell Biomedical Knowledge Hub (CBKH) that takes into account genes, drugs, diseases, anatomies, molecules, and symptoms, resulting in a KG with 2,231,297 entities of six types and 48, 678, 651 relations of eight types. To our best knowledge, there is no existing KG in the literature to model herbs, the contained chemicals, and their interactions with genes and diseases from the viewpoint of molecular medicine. Thus, our study aims to fill this gap.

The contributions of this study are summarized as follows:

• We propose a Herb ontology named HerbOnt composed of four entity types and five relation types to encode the interplay between herbs, chemicals, genes, and diseases.
• We develop a learning framework to automate the construction of HerbKG. We leverage an existing named entity recognition (NER) model and design a BERT-based model for relation extraction (RE) to annotate raw PubMed abstracts that are screened to match the subject of herbalism.
• To validate the RE model, we create a herb RE dataset with 3,526 human-annotated relations. BERT and two of its variants, SciBERT and BioBERT are evaluated on the herb RE dataset. In addition, two performance boosters, including a fine-tuning strategy and a substitution-based generative augmentation module, have been utilized for performance improvement. Our ablation studies show that the two boosters can bring consistent gains in the F1 score due to the additional domain knowledge injected into the models. The best model, the fine-tuned BioBERT that is further trained on the augmented dataset, can achieve an F1 score of 95.9%. The self-created herb RE dataset, with the evaluated models, can serve as a credible benchmark for future research.
• The proposed system has analyzed a total of 516,393 PubMed abstracts and identified 4,130 herbs, 6,331 chemicals, 2,187 diseases, and 2,641 genes, with 53,754 distinct relations, providing valuable domain knowledge in herbalism from the molecular perspective. In addition, the constructed HerbKG can support multiple downstream tasks like evidence-based graph queries and drug re-positioning. We have made the code publicly available at https://github.com/FeiYee/HerbKG, where an interactive tool and a system tutorial are also provided.

The rest of this paper is structured as follows. Section 2 provides the technical details of the proposed framework for HerbKG construction. Section 3 presents the experimental results and a discussion about several downstream tasks. Section 4 offers a summary of this study with limitations and future plans.

## 2 Materials and Methods

### 2.1 The Herb Ontology

**FIGURE 1:**
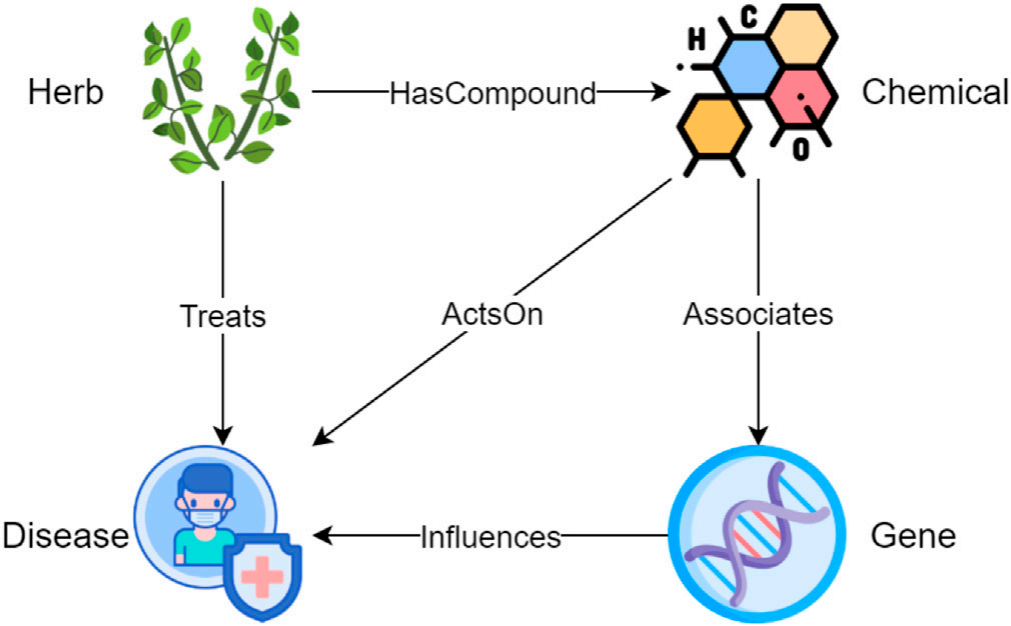
The proposed Herb ontology consists of four entity types and five relations. The four entity types are Herb, Chemical, Disease, and Gene; the five relation types are HerbHasCompoundChemical (HHC), HerbTreatsDisease (HTD), ChemicalActsOnDisease (CAD), ChemicalAssociatesGene (CAG), and GeneInfluencesDisease (GID).

In information sciences, an ontology shows the properties of a subject area and how they are related, by defining a set of categories and concepts that represent the subject (Guarino et al., 2009). To unify the terminology throughout the article, we use entity types to refer to categories and entities to refer to concepts that are instantiated from entity types. An ontology serves as a template for constructing a KG since only relations defined in the ontology can be added into the KG. As shown in Figure 1, HerbOnt consists of four entity types, including Herb, Chemical, Disease, and Gene, with five coarse-grained relation types, including HerbHasCompoundChemical (HHC), HerbTreatsDisease (HTD), ChemicalActsOnDisease (CAD), ChemicalAssociatesGene (CAG), and GeneInfluencesDisease (GID). A description of the entity types is as follows.

• Herbs in this study can be a part or produced from parts of a plant (either fresh or dried), including the leafy green or flowering parts, seeds, bark, roots and fruits. Examples include “abrus precatorius”, “ginkgo biloba”, “salvia officinalis”, and “cinnamomum cassia”.
• Chemicals refer to chemical compounds that can be used as medicine. In our study, we mainly focus on the chemicals extracted from herbs. Examples include “essential amino acids”, “isoflavanquinones”, “diphenhydramine”, and “abruquinone A”.
• A Disease refers to a particular abnormal condition that negatively affects the structure or function of all or part of an organism, and that is not due to any immediate external injury (Wikipedia contributors, 2004a). Examples include “anemia”, “otoconia”, “hypoosmotic swelling”, and “gastric ulcer”.
• A Gene refers to a basic unit of heredity and a sequence of nucleotides in DNA or RNA that encodes the synthesis of a gene product, either RNA or protein (Wikipedia contributors, 2004b). Examples include “caspase-3″, “AP-1″, “Bax”, and “cytochrome c”.

We also provide a description of the relation types below:

• An HHC describes a containment relation between a herb and a chemical, which is extracted from the herb. A herb may contain one or more chemicals that can be used for medical purposes. For example, cassia barks contains cinnamaldehyde.
• An HTD indicates that a herb has positive effect on the treatment of a disease.
• A CAD refers to a relation between a chemical and a disease. The effect of the chemical on the disease can be either positive or negative. CAD allows us to understand which chemical extracted from the herb causes what effect on the disease.
• A CAG describes an association between a chemical and a gene. For example, a study (Li et al., 2016) shows that cinnamaldehyde (a chemical) can inhibit the PI3K/Akt (a gene) signaling pathway, inducing apoptosis and affecting the biological behavior of human colorectal cancer cells.
• A GID indicates a connection between a gene and a disease. For example, a study (Lee et al., 2007) shows that AP-1 (a gene) inactivation can inhibit SW620 colon cancer (a disease) cell growth.

### 2.2 HerbKG Learning Tasks

Two learning tasks, including NER and RE, are involved in the construction of HerbKG. We provide a formal definition for each task as follows.

#### 2.2.1 NER Task

The goal of NER is to locate entity mentions in an input text and classify them into a set of pre-defined categories. Formally, let *s* = [*t*_1_, *t*_2_, *…* , *t*_*n*_] denote a sentence *s* with *n* tokens. Taking *s* as an input, an NER model outputs a list of tuples $<I_{s},I_{e},k>$ , each of which is an entity mention in *s*. Here, *I*_*s*_ and *I*_*e*_ specify the indices of the starting and ending tokens of an entity mention. Thus, both *I*_*s*_ and *I*_*e*_ are in [1, *n*], and *I*_*s*_ ≤ *I*_*e*_. Also, *k* belongs to a category set. In our study, *k* ∈ {*“Herb”*, *“Chemical”*, *“Disease”*, *“Gene”*}.

#### 2.2.2 RE Task

For the RE task, we choose to develop a medium-sized dataset, because there is no existing one that has the same ontology definition as HerbOnt. We formulate the learning problem as follows. Let $D_{RE}={\left\{\left(x_{i},y_{i}\right)\right\}}_{i=1}^{m}$ be the self-developed herb RE dataset with *m* examples. Each input *x*_*i*_ contains the title and content of an abstract and a pair of entities. The label *y*_*i*_ specifies the relation type of the entity pair in *x*_*i*_. The task is to train a model to predict the relation type given *x*_*i*_ as an input. It is noted that the problem belongs to document-level RE, where the head and tail entity mentions could span across multiple sentences in the abstract. Also, in the context of HerbKG, we have *y*_*i*_ ∈ {*“HHC”*, *“HTD”*, *“CAD”*, *“CAG”*, *“GID”*, *“Neg*.*“*}, in which the first five are positive relation types defined in HerbOnt, and “Neg.” represent the negative examples indicating a non-relation. Since not every entity pair of an abstract are related, it is essential to introduce negative examples into the dataset so that the model can be trained to make a distinction.

### 2.3 System Overview

**FIGURE 2:**
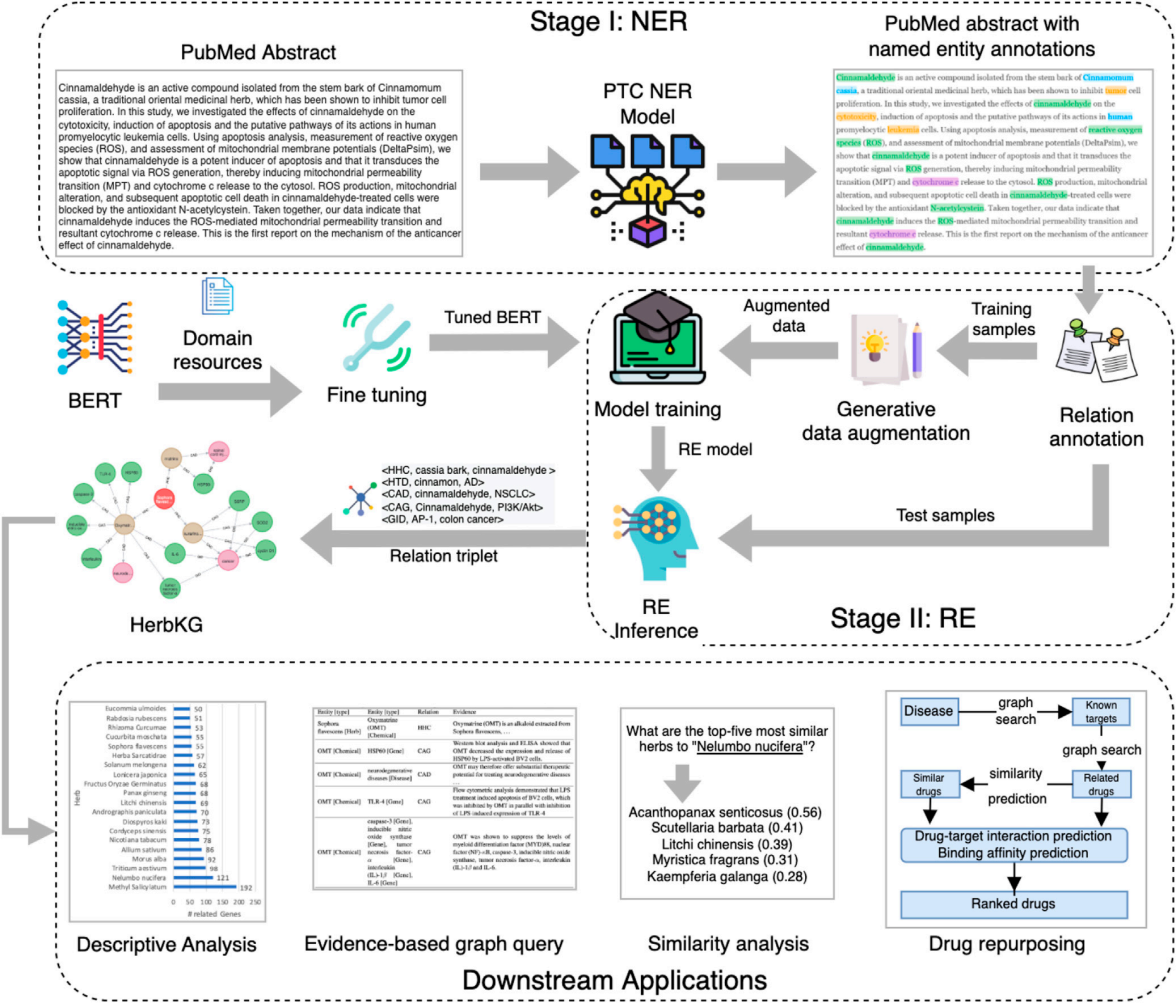
A two-stage learning framework for building HerbKG. Stage I is the NER task, which is done by the PTC NER model. Stage II is the RE task, which extracts relation triplets used to build the HerbKG. In addition, two boosting strategies have been utilized for performance improvement, including fine-tuning BERT on domain resources and a generative data augmentation method. The former aims to inject domain knowledge into the BERT model, while the latter can generate synthetic samples to enhance the training set. The constructed HerbKG can support multiple downstream applications, such as descriptive analysis, evidence-based graph query, similarity analysis, and drug repurposing.

Figure 2 shows a two-stage learning framework of building HerbKG. Since only a small fraction of PubMed articles are relevant to the subject of HerbKG, we develop a screening procedure to identify the matching abstracts. Specifically, only an abstract that contains a mention of either a herb or a chemical in a pre-defined domain vocabulary is considered as a match. In practice, it is straightforward to list the herbs and their contained chemicals for medicinal usage. This work is manually done by one of the authors with domain knowledge in herbal medicine. Each selected abstract is then passed through the PubTator Central (PTC) NER model (Wei et al., 2019), followed by a custom BERT-based RE model to produce a list of identified relation triplets, which are used for the HerbKG construction. In addition, two boosting strategies have been utilized for performance improvement, including fine-tuning BERT on domain resources and a generative data augmentation method. The former aims to inject domain knowledge into the BERT model, while the latter can generate synthetic samples to enhance the training set. The constructed HerbKG can support multiple downstream applications, such as descriptive analysis, evidence-based graph query, similarity analysis, and drug repurposing.

### 2.4 The PTC NER Model

**FIGURE 3:**
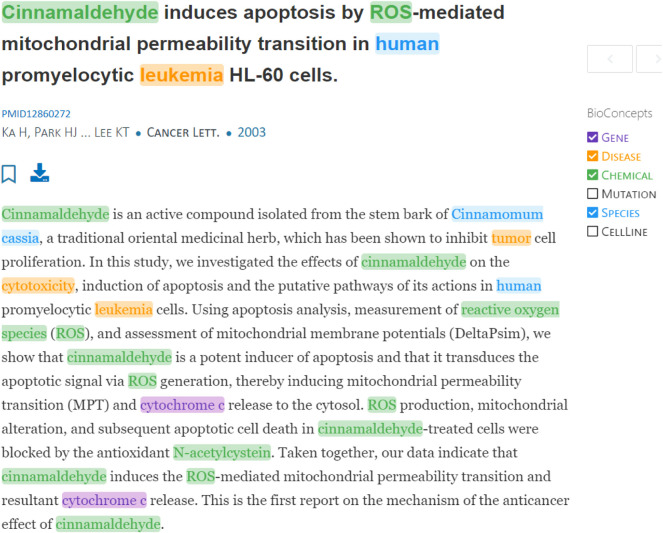
Bio-concept annotation through the PTC web interface for an article with PMID 12860272.

Numerous methods have been developed to solve NER. In this study, we adopt an existing model (Wei et al., 2019), referred to as PTC, proposed by Wei et al., who have provided an implementation hosted online at https://www.ncbi.nlm.nih.gov/research/pubtator/. PTC can identify and annotate six types of entities, including Gene, Disease, Chemical, Mutation, Species, and Cellline. At a high level, PTC works by feeding an article into a tagging module, which integrates a series of entity taggers including GNormPlus (Wei et al., 2015a), AB3P (Sohn et al., 2008), SimConcept (Wei et al., 2015b), tmVar 2.0 (Wei et al., 2018), SR4GN (Wei et al., 2012), TaggerOne (Leaman and Lu, 2016), and Cellosaurus (Bairoch, 2018). The tagging module also addresses several issues in bio-entity annotation, including abbreviation resolution, detection of composite/variant mentions, and entity normalization. The initial annotations are further processed by a concept disambiguation module to ensure that mentions referring to the same entity receive the same identifier. Figure 3 is a screenshot of bio-concept annotation using the PTC web interface for an article with PMID 12860272.

To adapt PTC to suit our needs, we disable the detection of mutations and celllines that are not defined in HerbOnt. Also, a detected species is annotated as a herb if it matches any entity in the pre-defined domain vocabulary. The top three sections in Table 1 display an annotated sample by PTC. These intermediate samples are further annotated by an RE model, which is discussed in the next subsection.

**TABLE 1:** A sample with annotation in the herb RE dataset.

Title	9848396 Cinnamaldehyde Inhibits Lymphocyte Proliferation and Modulates T-Cell Differentiation
Abstract	9848396 Two kinds of cinnamaldehyde derivative, 2′-hydroxycinnamaldehyde (HCA) and 2′-benzoxy-cinnamaldehyde (BCA), were studied for their immunomodulatory effects. These compounds were screened as anticancer drug candidates from stem bark of Cinnamomum cassia for their inhibitory effect on farnesyl protein transferase activity. Ras activation, which is accompanied with its farnesylation, has been known to be important in immune cell activation as well as in carcinogenesis. Treatment of these cinnamaldehydes to mouse splenocyte cultures induced suppression of lymphoproliferation following both Con A and LPS stimulation in a dose-dependent manner…
Entity Mentions	9848396 0 14 Cinnamaldehyde Chemical C012843
	9848396 127 151 2′-hydroxycinnamaldehyde Chemical C117567
	9848396 162 187 2′-benzoxy-cinnamaldehyde Chemical C117567
	9848396 322 339 Cinnamomum cassia Herb 119,260
	…
Relations	9848396 HHC 119260 D013390
	9848396 HHC 119260 C117567
	9848396 CAD D013390 C565232
	9848396 CAD C117567 C565232

### 2.5 A BERT-Based RE Model

As defined in Section 2.2.2, the RE task in this study is a multi-class classification problem aiming to predict the relation type, given an abstract and a pair of annotated entities. Since there is no existing dataset, we have developed a dataset and trained a BERT-based model for the herb RE task.

#### 2.5.1 Herb RE Dataset Development

**FIGURE 4:**
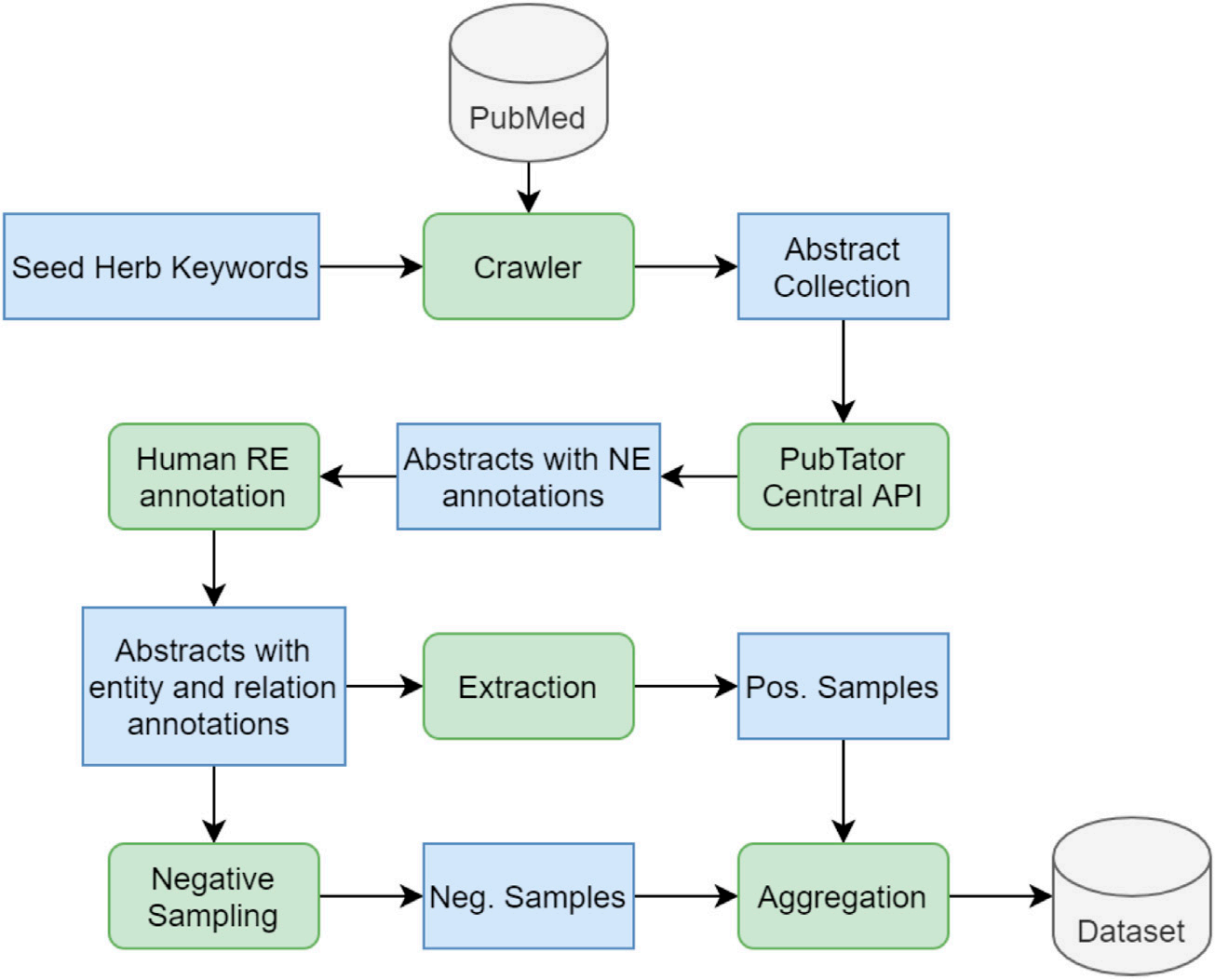
The development process of the Herb RE dataset. A crawler is adopted to search for a pre-defined set of herb keywords in the PubMed dataset, returning a collection of matching abstracts that are firstly annotated by the PTC for NER and then annotated by a human annotator for RE. Thereafter the positive examples can be directly extracted, and the negative examples can be obtained through negative sampling. The positive and negative samples are aggregated to form the final herb RE dataset. Blue boxes in the figure represent various types of data being processed along the pipeline, and green rounded boxes represent operations/functions applied on the data.

Figure 4 describes the dataset development process. First, we gather a collection of seed keywords of herb varieties that are commonly used in herbal medicine. We then use a self-developed crawler to search the PubMed dataset for the seed herb keywords. The search is only applied to the PubMed abstracts rather than the full texts, because an abstract contains the essential findings of a study; in most cases, the entities and their relations are clearly stated in an abstract, providing sufficient information that can be extracted to build our knowledge graph. The crawler is able to scrape a collection of relevant abstracts guided by the seed keywords. Next, the collected abstracts are fed into the PTC through its restful API at https://www.ncbi.nlm.nih.gov/research/pubtator/api.html (accessed on 7 July 2021). We display a complete sample with full annotation in Table 1, in which the first three rows, including Title, Abstract, and Entity Mentions, are generated by the PubTator API; the last row, Relations, is completed by a human annotator, who is a Ph.D. student in molecular biology with sufficient domain knowledge for the annotation task.

Each annotated abstract follows the PubTator format, as shown in Table 1, which divides a sample into four sections, each starting with the PubMed article ID of the abstract. The Title and Abstract sections that are directly extracted from the PubMed database. Each entity mention is a six-tuple with a specified sequence of PubMed ID, entity start position, end position, entity text, entity type, and entity ID. Similarly, each relation in the Relations section is a four-tuple sequence including the PubMed ID, the relation type, the head entity ID, and the tail entity ID.

Since we adopt BERT-based models for RE, the input can be split into two sentences, denoted by A and B. For our RE task, sentence A is a concatenation of the title and the abstract, and sentence B contains the head and tail entities, separated by a space. For each relation type, we need both positive and negative training examples. Also, each example in the dataset is a three-tuple, namely, sentence A, sentence B, and relation type that are separated by a tab. To simplify training, we group all negative examples together to create a new RE category, i.e., “Neg.“. Therefore, the RE type for each example is one of the elements in {*“HHC”*, *“HTD”*, *“CAD”*, *“CAG”*, *“GID”*, *“Neg*.*”*}, leading to a six-class classification problem. Table 2 shows five instances in the herb RE dataset.

**TABLE 2:** Instances in the herb RE dataset.

RE Type	Sentence A	Head Entity	Tail Entity
HHC	These results showed that cassia barks contained high contents of cinnamaldehyde …	cassia bark	cinnamaldehyde
HTD	…The extract of cinnamon bark contains potentially valuable antiamyloidogenic agents for the prevention and treatment of AD …	cinnamon	AD
CAD	…The present experiment showed that cinnamaldehyde dose-dependently depresses the proliferation of three types of NSCLC cells and induces cell apoptosis *in vitro* and *in vivo*…	cinnamaldehyde	NSCLC
CAG	…Cinnamaldehyde affects the biological behavior of human colorectal cancer cells and induces apoptosis via inhibition of the PI3K/Akt signaling pathway…	Cinnamaldehyde	PI3K/Akt
GID	…2-hydroxycinnamaldehyde inhibits SW620 colon cancer cell growth through AP-1 inactivation…	AP-1	colon cancer

Table 3 shows the stats of the herb RE dataset that contains a total of manually annotated 3,526 examples, split in the ratio of 3:1 to obtain the training and test sets. It is observed that the six classes are highly imbalanced. HHC and Neg. combined account for nearly 70% of all relations, while CAG and GID only take 1.3 and 3.1%, respectively. Thus, a performance metric (see Section 3.1.1) should be carefully chosen to properly deal with the class imbalance issue.

**TABLE 3:** Stats for the herb RE dataset.

	HHC	HTD	CAD	CAG	GID	Neg
Training	884	472	176	29	81	1,010
Test	346	201	68	16	29	224
Total	1,230	673	244	45	110	1,234
%	34.8	19.1	6.9	1.3	3.1	34.8

#### 2.5.2 BERT-Based Neural Architecture for RE

BERT (Devlin et al., 2018) is a transformer-based NLP model created and published in 2018 by Devlin et al. at Google. The original BERT model has two versions: Bert_*base*_ and Bert_*large*_. The former consists of a stack of 12 transformer encoders with 12 self-attention headers, and the latter includes 24 encoders with 16 self-attention headers. Each transformer encoder consists of a self-attention layer with multiple heads and a feed-forward layer. A self-attention head projects a sequence of input tokens into a latent space to capture the semantic dependency between the tokens. The output of the self-attention layer is normalized, aggregated, and passed through a feed-forward layer to produce a vector, namely, the hidden state, which is the output of the transformer encoder. The paths the tokens take to flow through the encoder can be partially executed in parallel, which allows the training and inference of the neural network to be accelerated. In this study, we adopt Bert_*base*_, which is pre-trained on BooksCorpus (Pechenick et al., 2015) (800M words) and on English Wikipedia (2,500M words). BERT adopts two unique pre-training techniques, i.e., masked-language modeling (MLM) and next sentence prediction (NSP), both of which are self-supervised. MLM works by randomly masking a fraction of tokens in the input sentence and training the model to predict the missing ones, while NSP trains BERT to predict the follow-up sentence given an input sentence. Both MLM and NSP aim to help BERT better understand the style of unstructured human language by optimizing the loss function of self-training and adjusting the model parameters. BERT has achieved the state-of-the-art (SOTA) performance in a variety of NLP tasks (Devlin et al., 2018) and yielded a spectrum of variants (Beltagy et al., 2019; Sanh et al., 2019; Lee et al., 2020).

**FIGURE 5:**
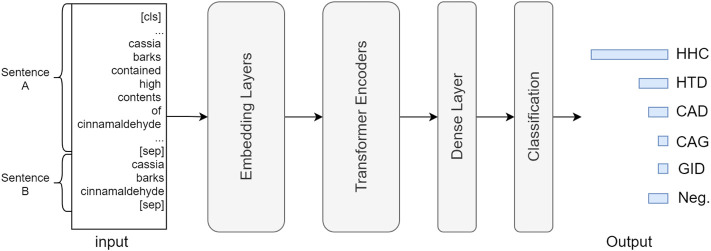
Neural architecture of BERT-based RE model. The input consists of sentence **(A,B)**, in which A is a concatenation of the title and the abstract content, and **(B)** includes the head and tail entity mentions. **(A)** (sep) token is placed at the end of each sentence as a separator. The input is processed through token, sentence, and positional embedding layers and then fed into a sequence of transformer encoder layers. Lastly, the output of the *N*th transformer passes a dense and a classification (softmax) layer to generate the prediction result.

Figure 5 describes the neural architecture of the BERT-based model for the herb RE task. Each input instance contains two strings, including sentences A and B, separated by a [sep] token. Sentence A is a concatenation of the title and abstract of a PubMed article, and sentence B contains an entity pair linked by a space character. The input passes through a stack of embedding layers for token, sentence, and positional embedding, transforming the original text to numeric vectors, which are further fed into a series of transformer encoders (Vaswani et al., 2017), where the neural parameters are updated via its unique self-attention mechanism. Lastly, the output of the *N*th transformer layer passes through a dense and classification (also softmax) layer to generate the prediction result, which is a six-dimensional normalized vector that encodes the confidence scores for each relation type. During training, the predicted and ground truth values are fed into a cross entropy loss function to calculate the loss for back-propagation.

We investigated BERT and two BERT variants, namely, BioBERT and SciBERT, to develop the benchmark models for the herb RE task.

• BioBERT (Lee et al., 2020) is a BERT variant pre-trained on PubMed articles for adapting the biomedical domain. BioBERT has been pre-trained on a large biomedical corpus with over a million PubMed articles, leading to superior performance in a variety of biomedical NLP tasks, compared to BERT and other pre-training models (Lee et al., 2020).
• SciBERT (Beltagy et al., 2019) is another BERT varient pre-trained on a corpus consisting of 1.14M full text scientific papers with 3.1B tokens collected from semanticscholar. org. As shown in (Beltagy et al., 2019), SciBERT has achieved SOTA performance in numerous NLP tasks in the scientific domain.

### 2.6 Performance Boosters

A big challenge faced by the RE task is the lack of training resources due to the high annotation cost that has to involve a human expert. Therefore, the RE model cannot absorb sufficient domain knowledge to always make the correct prediction. Two strategies, including fine-tuning and data augmentation, have been adopted to enhance the model’s learning capability in the context of herbal-molecular medicine.

#### 2.6.1 Fine-Tuning on Domain Resources

BERT, SciBERT, and BioBERT have been sufficiently pre-trained on different types of domain resources, namely, on general English texts, general scientific articles, and biomedical articles, respectively. Due to the disparity of domains, BERT and its two variants have learned different knowledge, which could lead to misclassification when applied to the domain of herbal-molecular medicine. Therefore, we conduct fine-tuning for all three models on the 516K PubMed abstracts to inject more domain knowledge into the models. The tuned models are then further trained to tackle the downstream RE task.

#### 2.6.2 Substitution-Based Generative Augmentation

**FIGURE 6:**
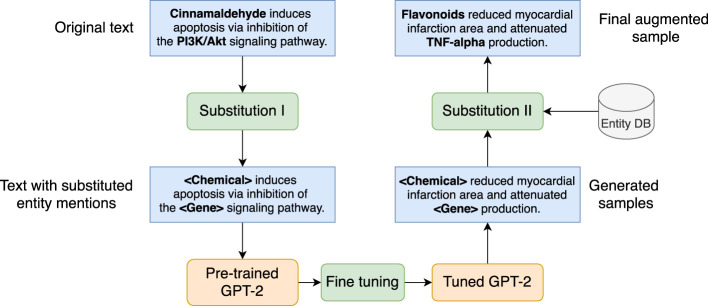
Substitution-based generative model for data augmentation. Blue boxes refer to text samples, green rounded boxes are procedures, and orange rounded boxes are models.

We employ a GPT-2-based generative model to generate synthetic samples to enhance the quantity and diversity of the training data. Figure 6 depicts the data augmentation mechanism, which includes the following steps.

• Corpus preparation. GPT-2 is a pre-trained generative model that can produce generic English sentences. To satisfy our requirements, GPT-2 needs to be fine-tuned on a corpus relevant to our RE task. Thus, the first is to prepare a corpus with textual resources that 1) are in the domain of herbal-molecular medicine and 2) present the entities and relations pre-defined in the herb ontology. Intuitively, we extract such sentences from the annotated dataset described in Section 2.5.1 based on one condition, namely, the sentence must contain at least a pair of entities that present one of the pre-defined relation categories. For example, the sentence “Cinnamaldehyde induces apoptosis via inhibition of the PI3K/Akt signaling pathway.” presents a CAG relation, is an ideal candidate to be added to the corpus. Since there are five relation types, five corpora are needed.
• Substitution I. For each sentence in each corpus, we apply a transformation by substituting each entity mention in the sentence with a type token. For example, the sentence in the above item becomes “[Chemical] induces apoptosis via inhibition of the [Gene] signaling pathway.” after the substitution. This step allows GPT-2 to focus on the semantic relations between generic entity types rather than particular entity mentions.
• Fine-tuning GPT-2. After substitution I, we send each corpus to fine-tune a GPT-2 model. The tuned GPT-2 model can generate sentences that are both semantically and syntactically similar to the ones in the input corpus. For instance, a generated sentence may look like “[Chemical] reduced myocardial infarction area and attenuated [Gene] production.”
• Substitution II. The generated samples are passed through another substitution block, which samples a pair of entities with known relations from an entity database (i.e., entity DB in the Figure) to replace the type token, namely, the placeholder, yielding the final augmented sample with the correct entity and relation annotation. The generated sentence from the previous item can become “Flavonoids reduced myocardial infarction area and attenuated TNF-alpha production.” which encodes a CAG relation between the entities in bold. The output of this module can be used to train the BERT-like models for the RE task.

## 3 Experiments and Results

All experiments were implemented using Python 3.6.7 and PyTorch 1.7.1 and conducted on a Windows 10 workstation with an i7-10875h CPU and a Tesla V100 16G GPU. We chose BERT base, which has 12 layers of encoders with a hidden size of 768, 12 attention heads, and 110M trainable parameters. As such, we choose the BERT base version for both SciBERT and BioBERT to conduct a comparable experiment.

### 3.1 RE Task Evaluation

We focus on the evaluation of the RE module for two reasons: 1) we adopted an existing NER model whose performance has been extensively evaluated in the original paper (Wei et al., 2019); 2) the outputs of the RE model are directly added into the HerbKG, which determines the quality of the KG.

#### 3.1.1 RE Performance Metric

Given a highly imbalanced RE dataset, accuracy is not an adequate metric since the model is encouraged to classify examples into the major classes and can still achieve a decent accuracy. A better metric to deal with class imbalance is the F1 score, which is defined as the harmonic mean of precision (Pre) and recall (Rec). Also, Pre and Rec are useful metrics since they are two inverse indicators of false alarms and missed instances, respectively. With the given true positives (TP), true negatives (TN), false positives (FP), and false negatives (FN), the definitions of Pre, Rec, and F1 are given in Eqs. 1–3. In addition, we choose the precision-recall area under the curve (PR AUC) as another important performance indicator, which is usually calculated by the average precision (AP) metric given in Eq 4, where precision *P* is expressed as a function of recall *R*, and AP is the average value of precision over the interval from *R* = 0 to *R* = 1. In other words, AP summarizes the PR curve as the weighted mean of precisions at each threshold, with the increase in recall from the previous threshold used as the weight, namely, *R*_*n*_ − *R*_*n*−1_ in Eq 4.

$$P=\frac{TP}{TP+FP}\times 100\%$$ (1)

$$R=\frac{TP}{TP+FN}\times 100\%$$ (2)

$$F1=2\times \frac{P\times R}{P+R}\times 100\%$$ (3)

$$AP=\int_{0}^{1}P\left(R\right)dr\approx \underset{n}{\sum }\left(R_{n}-R_{n-1}\right)P_{n}$$ (4)

#### 3.1.2 Hyperparameter Tuning

Since the three benchmark RE models adopt the same neural architecture, it is convenient to tune the models with the same set of hyperparameters in the same predefined ranges, which are given in Table 4. We tuned four hyperparameters, including epoch, learning rate, loss function, and optimizer, which are commonly used in prior efforts (Lee and Hsiang, 2019; Mosbach et al., 2020). For the number of epochs, we chose the odd numbers less than ten. Since the models have been extensively pre-trained, fine-tuning them for a downstream task would be fast (Zhu et al., 2021). For the learning rate, we chose four values including 0.0001, 0.0003, 0.001, 0.003, and 0.01. In practice, a large learning rate may bring difficulty in model convergence due to overshooting, and a small learning rate may lead to slow convergence (Goodfellow et al., 2016). For the loss function, we examined two options, including the standard cross entropy (CE), and the weighted CE. Since the class labels are imbalanced, using a weighted CE allows the algorithm to trade off recall and precision by up- or down-weighting the cost of a positive error relative to a negative error. Lastly, for the optimizer, we explored three options, including stochastic gradient descent (SGD), Adam, and SGD with Momentum. During training, SGD is computationally fast due to its frequent updates to the parameters; the Adam optimizer improves SGD by combining the AdaGrad and RMSProp algorithms to handle sparse gradients; adding momentum to SGD allows the optimization algorithm to accumulate the gradient used by the previous steps to calculate a better direction for the next step. A grid search is conducted based on the given ranges of hyperparameters to determine the optimal setting. Table 5 shows the best hyperparameters for each model obtained from the grid search using F1 as the selection criterion. It is observed that all three models demonstrate an F1 of over 92%. The best model, BioBERT, presents an F1 of 94.37%. A detailed breakdown of the results is discussed in the next subsection.

**TABLE 4:** Hyperparameter tuning range.

Hyperparameter	Tuned Range
Epoch	[1, 3, 5, 7, 9]
LR	[0.0001, 0.0003, 0.001, 0.003, 0.01]
Loss	[cross entropy, weighted cross entropy]
Optimizer	[SGD, Adam, Momentum]

**TABLE 5:** Optimal hyperparameter setting.

Hyperparameter	BERT	SciBERT	BioBERT
Epoch	5	3	3
LR	0.001	0.001	0.0003
Loss	CE	CE	CE
Optimizer	Adam	Adam	Adam
F1	0.9265	0.9344	**0.9473**

The highest F1 score is marked in bold.

#### 3.1.3 RE Performance Comparison

We report a performance comparison on the RE task for BERT, SciBERT, and BioBERT using their base models in Tables 6– 8 respectively. Each table is divided into two sections. The upper section is a confusion matrix that provides the exact RE classification results on the test set of the herb RE dataset, and the lower section presents the results in multiple metrics including TP, FP, FN, TN, Pre, Rec, and F1, which reflects the model performance from various aspects. We break down the result interpretation as follows.

• The overall ranking of the three models is BioBERT, SciBERT, and BERT, with an F1 of 94.7, 93.4, and 92.6%, respectively. This result is reasonable, since the three versions are pre-trained on corpora of different domains. BERT, SciBERT, and BioBERT are pre-trained on general English articles, scientific articles, and biomedical (i.e., PubMed) articles, presenting a narrower but more focused domain. It is clear that BioBERT has obtained extensive biomedical domain knowledge that well fits the herb RE task, leading to superior performance.
• The three minor relation types, namely, CAG, CAD, and GID, present worse performance due to insufficient training instances. Taking CAG as an example, there are only 16 instances in the test set. The BERT model throws two false alarms (the actual relations are HHC and HTD) and misclassifies three CAG instances into HHC (two cases) and CAD (one case), resulting in 13 instances correctly predicted. SciBERT fixes one error prediction (CAG misclassified as HHC) made by BERT. On the other hand, BioBERT eliminates all three false alarms, leading to a gain of 5.8% in F1, compared to BERT. Similar observations can be made for other relation types. In other words, BioBERT results in consistent performance gain across the individual relation types, demonstrating its superiority in the herb RE task.

**TABLE 6:** BERT performance.

	CAG	HHC	HTD	CAD	GID	Neg	Total
CAG	13	2	0	1	0	0	16
HHC	1	338	6	0	1	0	346
HTD	1	5	191	3	0	1	201
CAD	0	1	2	64	1	0	68
GID	0	0	0	4	25	0	29
Neg	0	2	1	1	0	220	224
TP	13	338	191	64	25	220	851
FP	2	10	9	9	2	1	33
FN	3	8	10	4	4	4	33
TN	838	513	660	787	826	631	4,255
Pre	86.7%	97.1%	95.5%	87.7%	92.6%	99.5%	93.2%
Rec	81.3%	97.7%	95.0%	94.1%	86.2%	98.2%	92.1%
F1	83.9%	97.4%	95.3%	90.8%	89.3%	98.9%	92.6%

**TABLE 7:** SciBERT performance.

	CAG	HHC	HTD	CAD	GID	Neg	Total
CAG	14	1	0	1	0	0	16
HHC	2	338	5	0	1	0	346
HTD	1	5	191	3	0	1	201
CAD	0	0	2	66	0	0	68
GID	0	0	0	4	25	0	29
Neg	0	2	1	1	0	220	224
TP	14	338	191	66	25	220	854
FP	3	8	8	9	1	1	30
FN	2	8	10	2	4	4	30
TN	840	516	663	788	829	634	4,270
Pre	82.4%	97.7%	96.0%	88.0%	96.2%	99.5%	93.3%
Rec	87.5%	97.7%	95.0%	97.1%	86.2%	98.2%	93.6%
F1	84.8%	97.7%	95.5%	92.3%	90.9%	98.9%	93.4%

**TABLE 8:** BioBERT performance.

	CAG	HHC	HTD	CAD	GID	Neg	Total
CAG	13	2	0	1	0	0	16
HHC	0	341	5	0	0	0	346
HTD	0	5	192	3	0	1	201
CAD	0	0	2	66	0	0	68
GID	0	0	0	4	25	0	29
Neg	0	2	1	0	0	221	224
TP	13	341	192	66	25	221	858
FP	0	9	8	8	0	1	26
FN	3	5	9	2	4	3	26
TN	845	517	666	792	833	637	4,290
Pre	100.0%	97.4%	96.0%	89.2%	100.0%	99.5%	97.0%
Rec	81.3%	98.6%	95.5%	97.1%	86.2%	98.7%	92.9%
F1	89.7%	98.0%	95.8%	93.0%	92.6%	99.1%	94.7%

**FIGURE 7:**
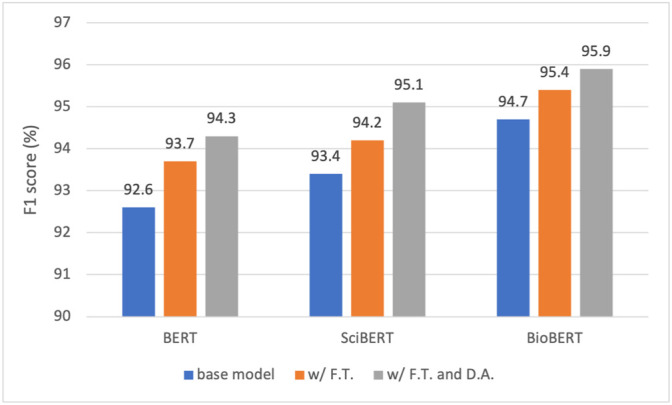
Performance comparison of BERT, SciBERT, and BioBERT in F1 score under three training settings: base model, with fine tuning (F. T.), and with F. T. and data augmentation (D. A.).

**FIGURE 8:**
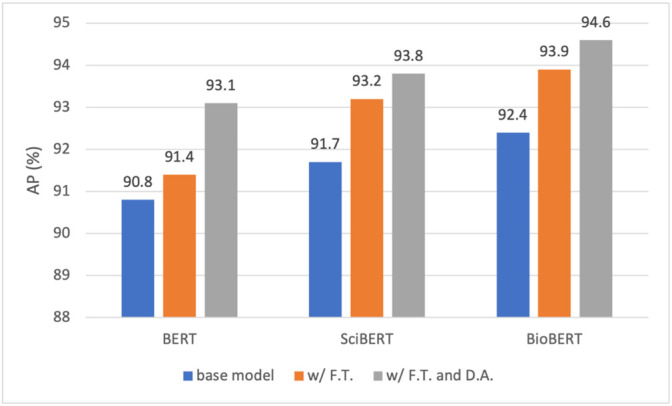
Performance comparison of BERT, SciBERT, and BioBERT in AP under three training settings: base model, with fine tuning (F. T.), and with F. T. and data augmentation (D. A.).

The results presented in Tables 6– 8 are from the base models of BERT, SciBERT, and BioBERT without any boosting strategy applied. Figures 7, 8 show the performance scores in F1 and AP for the three models. Specifically, for each model, we incrementally apply the two boosters, namely, fine-tuning and data augmentation, yielding six models. Our observations on the results are as follows.

• Both fine-tuning and data augmentation have demonstrated consistent performance gains for all three models, validating the effectiveness of the boosting strategies in the given context. The best model, namely, the fine-tuned BioBERT further trained on the augmented dataset, presents an F1 score of 95.9%.
• Fine-tuning has boosted the F1 by 1.1, 0.8, and 0.7%, for BERT, SciBERT, and BioBERT, respectively. It is observed that the gain reduces as the model moves from BERT to BioBERT, which can be explained from the perspective of the domain resources used for pre-training. BERT was pre-trained on generic English texts, which is distant from the domain of herbal-molecular medicine in this study. On the other hand, BioBERT has already been trained on PubMed articles, which are highly relevant to the domain for our task. Whereas SciBERT is somewhere in the middle. Therefore, fine-tuning BERT led to the most performance gain since more domain knowledge can be injected and transferred to the downstream task. In contrast, BioBERT does not benefit too much from the fine-tuning, because most syntactic and semantic patterns (i.e., domain knowledge) have been seen and learned during pre-training.
• Adding data augmentation on top of the three tuned models also brings consistent improvements, yielding a gain of 0.6, 0.9, and 0.5%, for BERT, SciBERT, and BioBERT, respectively. The gain is minor due to the strategy taken to generate the synthetic samples. As described in Section 2.6.2, a sentence is selected to fine-tune GPT-2 only if it contains two entity types with a known relation. As a result, the chosen sentences are generally short and only present intra-sentence relations. However, most of the hard cases are samples with inter-sentence relations. In other words, two entities may span multiple sentences to present a relation. Fortunately, these hard cases are rare in the abstracts of scientific papers. In fact, most authors tend to use concise and clear sentences to present scientific findings, which is good news for our RE task.
• Similar observations can be obtained from Figure 8 regarding the effects of fine-tuning and data augmentation on AP. The addition of fine-tuning brings up the AP by 0.6, 1.5, and 1.5% for the three base models, and the addition of data augmentation leads to a gain of 1.7, 0.6, and 0.7% for the three fine-tuned models. The gains have been consistent across for both metrics with all three models, validating the efficacy of the two boosters.

#### 3.1.4 Token Importance Evaluation

**FIGURE 9:**
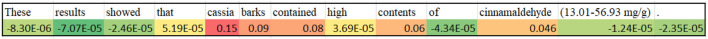
An examination of token importance in the determination of a relation type. The example shows in the figure is correctly classified by the BioBERT-based RE model, which outputs a triplet (“cassia bark”, “cinnamaldehyde”, “HHC”) as an entry in the HerbKG.

In this section, we discuss how BioBERT is trained to learn the relations using several qualitative results to gain a deeper understanding on the impacts of individual tokens on the determination of a relation. The process is as follows. For each instance in the test set, only sentences that contain both head and tail entities are kept and saved in a list. Let *s* = [*t*_1_, *t*_2_, *…* , *t*_*n*_] denote an extracted sentence with *n* tokens *t*_1_, *…* , *t*_*n*_. Our goal is to calculate a score that measures the impact of each individual token on the relation of an entity pair. Specifically, we first pass *s* through the fine-tuned BioBERT model to obtain a score denoted by c_*_, which represents the probability that *s* is classified into the correct relation type. Thereafter a loop is employed to iterate through *s* token by token. For the *i*th iteration, token *t*_*i*_ is replaced by a meaningless token *t*′, and the modified sentence is passed through the same BioBERT model once again to obtain another score denoted by *c*_*i*_. The score difference, denoted by *d*_*i*_ = c_*_ − *c*_*i*_, reflects the importance of token *t*_*i*_. In other words, the larger the *d*_*i*_, the more greatly the confidence score drops, thus, the more important *t*_*i*_ is. Figure 9 shows an example where *d*_*i*_ is obtained for each token *i*. The sentence is extracted from an instance in the test set and expresses a HHC relation between “cassia bark” and “cinnamaldehyde”. It is observed that tokens that receive high importance scores fall into two categories: 1) tokens such as “cassia”, “barks”, and “cinnamaldehyde” are parts of the entities that are obviously important; 2) tokens such as “contained” and “contents” are the keywords that semantically determine the relation type. For the latter case, it is noted that BioBERT can effectively learn and quantify the impacts of tokens in a given instance, demonstrating its superior capability of semantic reasoning.

### 3.2 The Constructed HerbKG

**FIGURE 10:**
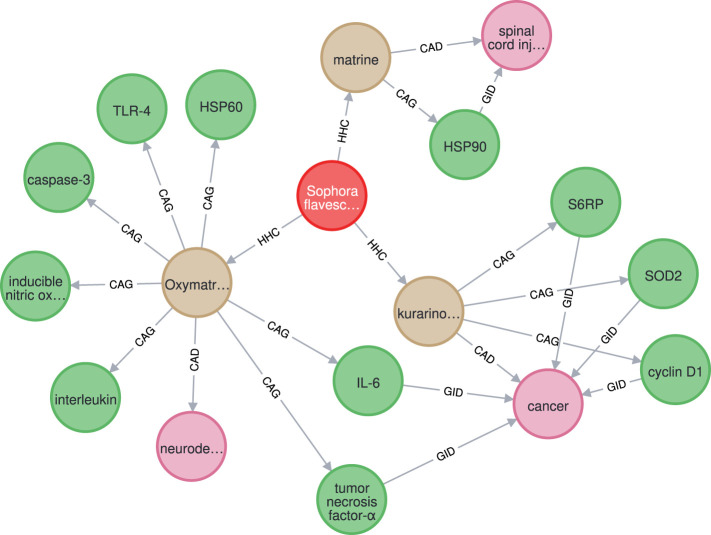
The extracted graph for herb “Sophora flavescens” is a subgraph of HerbKG, in which Herb, Chemical, Gene, and Disease entities are marked in red, tan, green, and pink, repsectively.

The proposed system analyzed a total of 516,393 PubMed abstracts and identified 4,130 herbs, 6,331 chemicals, 2,187 diseases, and 2,641 genes, with 53,754 distinct relations, including 19,872 HHC, 13,627 HTD, 9,984 CAD, 3,353 CAG, and 6,918 GID relations. A subgraph of HerbKG (stored using the Neo4j graph database (Webber, 2012)) is shown in Figure 10, which consists of one herb entity, three chemical, eleven gene, and three disease entities. Also, the subgraph includes three HHC, eleven CAG, six GID, and three CAD relations. It is noted that only three abstracts were processed through the proposed learning pipeline to generate this subgraph.

### 3.3 Downstream Applications

This subsection covers four categories of downstream applications with several case studies to demonstrate the potential of HerbKG to provide data-driven and evidence-based knowledge support in pharmacology.

#### 3.3.1 Descriptive Analysis

**FIGURE 11:**
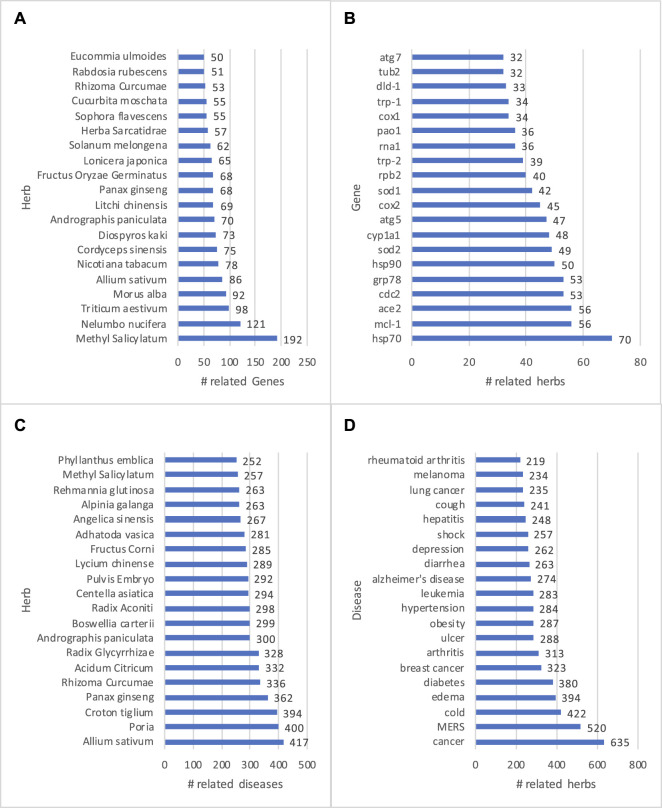
Examples of descriptive analysis are shown. The HerbKG can provide the top-ranked herbs with the most related genes **(A)**, the genes regulated by the most herb extracts **(B)**, the herbs that can treat the most diseases **(C)**, and the most treated diseases by herbs **(D)**.

An advantage of a KG is that data, as stored in entities and relations, can be easily visualized and presented to end users. Thus, knowledge visualization has been a basic feature for KG-based applications (Yu et al., 2017; Liu et al., 2018; Wise et al., 2020; Zheng et al., 2020). In addition, descriptive analysis, which helps describe, show or summarize data points, is desired in a dashboard interface that allows a user to quickly grasp a big picture of data. Statistical results can be customized, presented, and visualized (Su et al., 2021; Zheng et al., 2021). The core mission of HerbKG is to investigate the molecular mechanism of herbal medicine. Therefore, it is crucial to understand the physiological behavior of herbs in the treatment of diseases by regulating gene expression/function. At a high level, the HerbKG can provide the top-ranked herbs with the most related genes (Figure 11A, the genes regulated by the most herb extracts (Figure 11B, the herbs that can treat the most diseases (Figure 11C), and the most treated diseases by herbs (Figure 11D). It is observed that five herbs, including Methyl Salicylatum, Allium sativum, Andrographis Paniculata, Panax Ginseng, and Rhizoma Curcumae, appear in both list (a) and (c), indicating that the extracted chemicals from these herbs have been extensively experimented to validate their effects on the diseases at the molecular level. Also, it is found that three heat shock proteins, namely HSP70, HSP90, and GRP78, are among the top-ten genes in list (b). Heat-related proteins have shown significance in clinical trials, especially in cancer treatment. Other top-ranked genes include MCL-1 (related to Myeloid Leukemia), ACE2 (related to human coronavirus), SOD2 (related to idiopathic cardiomyopathy, premature aging, sporadic motor neuron disease, and cancer), and so on. In addition, the top-ranked diseases in list (d) include several deadliest diseases, such as various types of cancer, diabetes, Alzheimer’s disease, and lower respiratory infections (e.g., MERS). Meanwhile, herbs are used to treat a wide range of common diseases, such as cold, obesity, hypertension, cough, and diarrhea. In all, these statistical results can be displayed in a dashboard to help users gain a high-level understanding of the commonly studied herbs and their related genes and diseases.

#### 3.3.2 Evidence-Based Graph Queries

KGs that are built from scientific articles are supported by the research findings in the source articles. Since users of such KGs could be researchers, doctors, or clinical practitioners, providing an evidence for each query that points to the original source is a huge advantage. With this feature, users can be more convinced by the information found in the KG and can be easily re-directly to the first-hand resource (Miao et al., 2018; Zheng et al., 2021). In this study, each extracted relation in HerbKG is based on a sentence in an abstract. The sentence where both entities appear becomes the evidence supporting the relation. For example, Table 9 shows a collection of relations learned from an abstract (PMID27882228), which is a study that investigated Oxymatrine (OMT), a component of Sophora flavescens, and its potential treatment for neurodegenerative diseases via the regulation of a set of genes. The test was done using our best model and achieved a satisfactory result, except that the CAG relation between OMT and myeloid differentiation factor (MYD)88 (last row of the table) was not detected due to the fact that the PubTator-based NER model failed to classify it as a gene in the first place. Each sentence that contains a detected relation was marked as a piece of evidence, and the relation is reinforced if it is supported by multiple evidence from different articles, meaning that the relation has been validated by more than one study and becomes more convinced. With this setup, a wide range of graph queries can be made. Since HerbKG is stored in a Neo4j graph database, a question should be first translated into a query statement in Cypher and sent to the Neo4j database engine; then a resulting subgraph is returned. For example, if our query were “find the top three most studied chemicals of Sophora flavescens and their related entities,” the result would be the entire graph in Figure 10.

**TABLE 9:** Case study: herbal-molecular knowledge extracted from an abstract (PMID27882228).

Entity [Type]	Entity [Type]	Relation	Evidence
Sophora flavescens [Herb]	Oxymatrine (OMT) [Chemical]	HHC	Oxymatrine (OMT) is an alkaloid extracted from Sophora flavescens...
OMT [Chemical]	HSP60 [Gene]	CAG	Western blot analysis and ELISA showed that OMT decreased the expression and release of HSP60 by LPS-activated BV2 cells
OMT [Chemical]	neurodegenerative diseases [Disease]	CAD	OMT may therefore offer substantial therapeutic potential for treating neurodegenerative diseases ...
OMT [Chemical]	TLR-4 [Gene]	CAG	Flow cytometric analysis demonstrated that LPS treatment induced apoptosis of BV2 cells, which was inhibited by OMT in parallel with inhibition of LPS-induced expression of TLR-4
OMT [Chemical]	caspase-3 [Gene], inducible nitric oxide synthase [Gene], tumor necrosis factor-*α* [Gene], interleukin (IL)-1*β* [Gene], IL-6 [Gene]	CAG	OMT was shown to suppress the levels of myeloid differentiation factor (MYD)88, nuclear factor (NF)-*κ*B, caspase-3, inducible nitric oxide synthase, tumor necrosis factor-*α*, interleukin (IL)-1*β* and IL-6

#### 3.3.3 Similarity Analysis

Similarity analysis in a KG is useful analytical method that measures the entity-entity or relation-relation similarity. Wang et al. utilize the Pearson correlation (Benesty et al., 2009) to represent the semantic similarity of herbs (Wang et al., 2019). Recent graph neural network (GNN) models facilitate this analysis by encoding a collection of relevant features into a node/relation embedding, which can be directly used for similarity calculation (Shen et al., 2019; Wise et al., 2020; Zheng et al., 2021). For example, Fokoue et al. adopt a GNN to compute drug similarity, which is used as a feature to predict drug-drug interaction. A unique value provided by HerbKG is the interplay between a herb-extracted chemical and a gene, which affects a disease. To quantify the similarity between two herbs in their biological functions, we focus on two aspects, namely, the shared set of genes they regulate and the shared set of diseases they may treat. Let $S_{G}^{i}$ and $S_{D}^{i}$ denote the set of genes herb extract *i* can regulate and the set of diseases *i* may treat, respectively. The similarity between two herb extracts *i* and *j* is given as follows.

$$sim\left(i,j\right)=\frac{1}{2}\left(\frac{S_{G}^{i}⋂\overset{j}{\underset{G}{S}}}{S_{G}^{i}⋃\overset{j}{\underset{G}{S}}}+\frac{S_{D}^{i}⋂\overset{j}{\underset{D}{S}}}{S_{D}^{i}⋃\overset{j}{\underset{D}{S}}}\right)$$

Intuitively, the more related genes and diseases two chemicals share, the more similar they are. With this idea, we performed similarity analysis for “Nelumbo nucifera”, and found the top-five most similar herbs as follows: Acanthopanax senticosus (0.56), Scutellaria barbata (0.41), Litchi chinensis (0.39), Myristica fragrans (0.31), Kaempferia galanga (0.28), where the values in the brackets indicate the similarity scores. Understanding the bio-function similarity between herbs is an important first step for drug repositioning, discussed in the following subsection.

#### 3.3.4 Drug Repurposing

Drug repurposing (or repositioning) aims to discover an existing drug’s new medical indications outside of the scope of its original usage (Zhu et al., 2020). KG-based drug repurposing aims to discover potential drug-target or drug-disease relations that do not currently exist in the KG (Boudin, 2020). Existing efforts have leveraged KGs to identify drugs for the treatment of Covid-19 (Al-Saleem et al., 2021) and rare diseases (Sosa et al., 2019). It is also noted that a drug-disease relation may be either direct or indirect, investigated in (Zhu et al., 2020), where different types of path between a drug and a disease are considered. On HerbKG, drug repositioning can be transformed to a link prediction problem that predicts a potential association between a chemical and a disease, which are not previously connected. We could use the following steps. First, a disease, say, the Parkinson’s disease is selected. Second, we locate the Parkinson’s disease in HerbKG as well as the its related herbs, chemicals, and genes. Third, the identified genes serve as starting point to find the associated chemicals that are not directly connected to the disease in HerbKG. These chemicals may be considered as drug candidates. However, if no such chemicals can be found, a signature matching process can be employed by comparing the biological profile (including information about structure, genetic and disease association, adverse effect, and graph properties, etc.) of a drug with that of another drug that is known to be used for treating the disease. In fact, matching is an operation that measures the similarity between chemicals, which can be done *via* machine learning. Specifically, such a learning model takes as input a chemical’s biological profile and the disease name and predicts a score that indicates the likelihood that the chemical can treat the disease. The current version of HerbKG has only encoded the knowledge of genetic and disease association, whereas more profile information for chemicals is needed to build an accurate model. In addition to the chemical-disease association, a more effective approach is to predict the chemical-gene interaction, given that most diseases are known to be caused by specific genes with aberrant expression patterns. Therefore, the top-ranked chemical-gene pairs can be generated by the predictive model and used for further validation to support the new usage of a drug.

## 4 Discussion

Recent advances have witnessed the prosperity of KGs, which can effectively represent knowledge in the physical world. Each KG is a semantic network that stores a collection of triplets, each of which encodes the relation between a pair of entities. KGs can support a wide range of applications, such as knowledge reasoning, information retrieval, question answering, and visualization. Also, domain specific KGs have received numerous interests from domain professionals and practitioners. A typical example is a biomedical KG, which allows doctors and researchers to mine and discover interplay between bio-entities, potentially accelerating the efficiency and improving the accuracy of current clinical practice.

Traditional medicine that uses herbs for health care has been existing for over five thousand years. However, herbalism has been criticized for its insufficiently verified efficacy and safety in modern medicine research. Current herbal KGs mainly focus on the diagnosis and treatment side of herbalism that explore relations between herbs, symptoms, and treatments, rather than investigate it from the view point of molecular medicine. In the past decades, more researchers adopt modern approaches in molecular medicine to study how herbs and their extracted contents affect the biological functions of human body. Our investigation shows that this type of researches have not been extensively utilized in the KG community, opening a promising research direction.

Our study aims to construct a KG to bridge herbal and molecular medicine. We propose HerbKG with four entities, namely, herbs, chemicals that are extracted from the herbs, diseases that can be treated by herb contents, and genes that are affected by the chemicals. Six relation types are defined to model the interplay between the entities. We develop a systematic framework to automate the construction of HerbKG by extracting relational triplets from PubMed abstracts. The proposed framework adopts an existing NER (i.e., PTC) model and a custom BERT-based RE model. The RE model is validated on a self-created herb RE dataset and demonstrates superior performance. The resulting HerbKG, after analyzing over 516K abstracts, is populated with 53,754 relations, offering valuable domain knowledge in herbalism from the molecular perspective.

A key challenge for supervised learning in a domain-specific task is the lack of abundant training resource. This challenge is addressed with two unsupervised strategies in our work. The first strategy, fine-tuning on domain resources, is to encourage the BERT model to learn more domain knowledge. The second strategy, substitution-based generative augmentation, aims to generate synthetic training samples based on the existing expert-annotated ones. The mixing of supervised and unsupervised learning paradigms brings new opportunities to tackle the problem of KG construction.

This study has the following limitations that will be addressed in future work. First, the RE task can be made more fine-grained. For example, the role of a chemical played in regulating a gene’s activity or function can be divided into several subclasses such as inhibitor, activator, antagonist, and agonist, etc. Fine-grained relations can enhance the knowledge granularity encoded by HerbKG and better support the downstream applications. Another direction is to explore existing data resources. After all, several well-known benchmarks that model either drug-drug, chemical-protein, or chemical-disease relations have been studied and can be utilized in model training. Lastly, more advanced downstream applications in drug discovery can be developed. Opportunities are twofold. One idea is to adopt a GNN model to better encode a wider spectrum of data properties, such as multi-omics, molecular structural, and graph properties, for entities and relations in HerbKG and facilitate the link prediction tasks like drug repurposing and target inference. Also, predicting the effect of drug combination could be a significant task that comes with a unique advantage in herbal medicine since drug compound has been a typical way of prescription in traditional herblism. As such, abundant training data can be gathered to train learning models.

## Data Availability

The datasets presented in this study can be found in online repositories. The names of the repository/repositories and accession number(s) can be found below: https://github.com/FeiYee/HerbKG.

## References

[B1] Al-Saleem J. Granet R. Ramakrishnan S. Ciancetta N. A. Saveson C. Gessner C. (2021). Knowledge Graph-Based Approaches to Drug Repurposing for Covid-19. J. Chem. Inf. Model. 61, 4058–4067. 10.1021/acs.jcim.1c00642 34297570

[B2] Auer S. Bizer C. Kobilarov G. Lehmann J. Cyganiak R. Ives Z. (2007). “Dbpedia: A Nucleus for a Web of Open Data,” in The Semantic Web (Berlin, Heidelberg: Springer), 722–735. 10.1007/978-3-540-76298-0_52

[B3] Babu P. S. Prabuseenivasan S. Ignacimuthu S. (2007). Cinnamaldehyde-A Potential Antidiabetic Agent. Phytomedicine 14, 15–22. 10.1016/j.phymed.2006.11.005 17140783

[B4] Bairoch A. (2018). The Cellosaurus, a Cell-Line Knowledge Resource. J. Biomol. Tech. 29, 25–38. 10.7171/jbt.18-2902-002 29805321 PMC5945021

[B5] Beltagy I. Lo K. Cohan A. (2019). Scibert: A Pretrained Language Model for Scientific Text. arXiv preprint arXiv:1903.10676.

[B6] Benesty J. Chen J. Huang Y. Cohen I. (2009). “Pearson Correlation Coefficient,” in Noise Reduction in Speech Processing (Berlin, Heidelberg: Springer), 1–4. 10.1007/978-3-642-00296-0_5

[B7] Bollacker K. Evans C. Paritosh P. Sturge T. Taylor J. (2008). “Freebase: A Collaboratively Created Graph Database for Structuring Human Knowledge,” in Proceedings of the 2008 ACM SIGMOD International Conference on Management of Data, Vancouver, Canada, June 9–12, 2008, 1247–1250.

[B8] Boudin M. (2020). “Computational Approaches for Drug Repositioning: Towards a Holistic Perspective Based on Knowledge Graphs,” in Proceedings of the 29th ACM International Conference on Information & Knowledge Management, October 2020, 3225–3228.

[B9] Brackman G. Defoirdt T. Miyamoto C. Bossier P. Van Calenbergh S. Nelis H. (2008). Cinnamaldehyde and Cinnamaldehyde Derivatives Reduce Virulence in Vibrio Spp. By Decreasing the Dna-Binding Activity of the Quorum Sensing Response Regulator Luxr. BMC Microbiol. 8, 1–14. 10.1186/1471-2180-8-149 18793453 PMC2551610

[B10] Burstein H. J. Gelber S. Guadagnoli E. Weeks J. C. (1999). Use of Alternative Medicine by Women with Early-Stage Breast Cancer. N. Engl. J. Med. 340, 1733–1739. 10.1056/nejm199906033402206 10352166

[B11] Chen X. Jia S. Xiang Y. (2020). A Review: Knowledge Reasoning Over Knowledge Graph. Expert Syst. Appl. 141, 112948. 10.1016/j.eswa.2019.112948

[B12] Devlin J. Chang M.-W. Lee K. Toutanova K. (2018). Bert: Pre-Training of Deep Bidirectional Transformers for Language Understanding. arXiv preprint arXiv:1810.04805.

[B13] Egede L. E. Ye X. Zheng D. Silverstein M. D. (2002). The Prevalence and Pattern of Complementary and Alternative Medicine Use in Individuals with Diabetes. Diabetes Care 25, 324–329. 10.2337/diacare.25.2.324 11815504

[B14] Ernst P. Meng C. Siu A. Weikum G. (2014). “Knowlife: a Knowledge Graph for Health and Life Sciences,” in 2014 IEEE 30th International Conference on Data Engineering, Chicago, IL, March 31–April 4, 2014 (IEEE), 1254–1257. 10.1109/icde.2014.6816754

[B15] Gong Z. Zhang N. He J. (2021). “Kgrn: Knowledge Graph Relational Path Network for Target Prediction of Tcm Prescriptions,” in International Conference on Intelligent Computing, Xi’an, China, August 7–11, 2021 (Springer), 148–161. 10.1007/978-3-030-84532-2_14

[B16] Goodfellow I. Bengio Y. Courville A. (2016). Deep Learning. Cambridge, MA: MIT press.

[B17] Guarino N. Oberle D. Staab S. (2009). “What Is an Ontology?,” in Handbook on Ontologies. Editors Staab S. Studer R. (Berlin, Heidelberg: Springer), 1–17. 10.1007/978-3-540-92673-3_0

[B18] Kerdjoudj F. Curé O. (2015). Rdf Knowledge Graph Visualization from a Knowledge Extraction System. arXiv preprint arXiv:1510.00244.

[B19] Leaman R. Lu Z. (2016). Taggerone: Joint Named Entity Recognition and Normalization with Semi-markov Models. Bioinformatics 32, 2839–2846. 10.1093/bioinformatics/btw343 27283952 PMC5018376

[B20] Lee C. W. Lee S. H. Lee J. W. Ban J. O. Lee S. Y. Yoo H. S. (2007). 2-hydroxycinnamaldehyde Inhibits Sw620 colon Cancer Cell Growth through Ap-1 Inactivation. J. Pharmacol. Sci. 104, 19–28. 10.1254/jphs.fp0061204 17510524

[B21] Lee J. Yoon W. Kim S. Kim D. Kim S. So C. H. (2020). Biobert: A Pre-Trained Biomedical Language Representation Model for Biomedical Text Mining. Bioinformatics 36, 1234–1240. 10.1093/bioinformatics/btz682 31501885 PMC7703786

[B22] Lee J.-S. Hsiang J. (2019). Patentbert: Patent Classification with fine-tuning a Pre-trained Bert Model. arXiv preprint arXiv:1906.02124.

[B23] Li J. Teng Y. Liu S. Wang Z. Chen Y. Zhang Y. (2016). Cinnamaldehyde Affects the Biological Behavior of Human Colorectal Cancer Cells and Induces Apoptosis via Inhibition of the Pi3k/akt Signaling Pathway. Oncol. Rep. 35, 1501–1510. 10.3892/or.2015.4493 26677144

[B24] Liu Z. Peng E. Yan S. Li G. Hao T. (2018). “T-Know: A Knowledge Graph-Based Question Answering and Infor-Mation Retrieval System for Traditional Chinese Medicine,” in Proceedings of the 27th International Conference on Computational Linguistics: System Demonstrations, Chongqing, China, Oct 12–14, 2018 (Santa Fe, New Mexico: Association for Computational Linguistics), 15–19.

[B25] Mahdisoltani F. Biega J. Suchanek F. (2014). “Yago3: A Knowledge Base from Multilingual Wikipedias,” in 7th Biennial Conference on Innovative Data Systems Research (CIDR Conference), Asilomar, CA, January 4 – 7, 2014.

[B26] Miao F. Liu H. Huang Y. Liu C. Wu X. (2018). “Construction of Semantic-Based Traditional Chinese Medicine Prescription Knowledge Graph,” in 2018 IEEE 3rd Advanced Information Technology, Electronic and Automation Control Conference (IAEAC), Chongqing, China, October 12 – 14, 2018, 1194–1198. 10.1109/iaeac.2018.8577236

[B27] Mosbach M. Andriushchenko M. Klakow D. (2020). On the Stability of fine-tuning Bert: Misconceptions, Explanations, and strong Baselines. arXiv preprint arXiv:2006.04884.

[B28] Navigli R. Ponzetto S. P. (2012). Babelnet: The Automatic Construction, Evaluation and Application of a Wide-Coverage Multilingual Semantic Network. Artif. Intelligence 193, 217–250. 10.1016/j.artint.2012.07.001

[B29] Pechenick E. A. Danforth C. M. Dodds P. S. (2015). Characterizing the Google Books Corpus: Strong Limits to Inferences of Socio-Cultural and Linguistic Evolution. PloS One 10, e0137041. 10.1371/journal.pone.0137041 26445406 PMC4596490

[B30] Roozbeh J. Hashempur M. H. Heydari M. (2013). Use of Herbal Remedies Among Patients Undergoing Hemodialysis. Iran J. Kidney Dis. 7, 492–495. 24241097

[B31] Saha A. Pahuja V. Khapra M. M. Sankaranarayanan K. Chandar S. (2018). “Complex Sequential Question Answering: Towards Learning to converse over Linked Question Answer Pairs with a Knowledge Graph,” in Thirty-Second AAAI Conference on Artificial Intelligence, New Orleans, Louisiana, February 2 - 7, 2018.

[B32] Sanh V. Debut L. Chaumond J. Wolf T. (2019). Distilbert, a Distilled Version of Bert: Smaller, Faster, Cheaper and Lighter. arXiv preprint arXiv:1910.01108.

[B33] Shen Y. Yuan K. Dai J. Tang B. Yang M. Lei K. (2019). Kgdds: A System for Drug-Drug Similarity Measure in Therapeutic Substitution Based on Knowledge Graph Curation. J. Med. Syst. 43, 1–9. 10.1007/s10916-019-1182-z 30834481

[B34] Sohn S. Comeau D. C. Kim W. Wilbur W. J. (2008). Abbreviation Definition Identification Based on Automatic Precision Estimates. BMC bioinformatics 9, 402. 10.1186/1471-2105-9-402 18817555 PMC2576267

[B35] Somé B. M. J. Bordea G. Thiessard F. Diallo G. (2019). “Enabling West African Herbal-Based Traditional Medicine Digitizing: the Watrimed Knowledge Graph,” in MEDINFO 2019: Health and Wellbeing e-Networks for All (Amsterdam, Netherlands: IOS Press), 1548–1549. 10.3233/SHTI190528 31438225

[B36] Sosa D. N. Derry A. Guo M. Wei E. Brinton C. Altman R. B. (2019). A Literature-Based Knowledge Graph Embedding Method for Identifying Drug Repurposing Opportunities in Rare Diseases. Pac. Symp. Biocomput 25, 463–474. 10.1142/9789811215636_0041 PMC6937428 31797619

[B37] Su C. Hou Y. Guo W. Chaudhry F. Ghahramani G. Zhang H. (2021). Cbkh: The cornell Biomedical Knowledge Hub. medRxiv. 10.1101/2021.03.12.21253461

[B38] Szelenyi I. Brune K. (2002). Herbal Remedies for Asthma Treatment: Between Myth and Reality. Drugs Today 38, 265. 10.1358/dot.2002.38.4.668337 12532195

[B39] Vaswani A. Shazeer N. Parmar N. Uszkoreit J. Jones L. Gomez A. N. (2017). “Attention Is All You Need,” in Advances in Neural Information Processing Systems (Curran Associates Inc), 5998–6008.

[B40] Wang Q. Mao Z. Wang B. Guo L. (2017). Knowledge Graph Embedding: A Survey of Approaches and Applications. IEEE Trans. Knowl. Data Eng. 29, 2724–2743. 10.1109/tkde.2017.2754499

[B41] Wang X. Zhang Y. Wang X. Chen J. (2019). “A Knowledge Graph Enhanced Topic Modeling Approach for Herb Recommendation,” in International Conference on Database Systems for Advanced Applications, Chiang Mai, Thailand, April 2019 (Springer), 709–724. 10.1007/978-3-030-18576-3_42

[B42] Webber J. (2012). “A Programmatic Introduction to Neo4j,” in Proceedings of the 3rd annual conference on Systems, programming, and applications: software for humanity, Tucson, AZ, October 12 - 15, 2012, 217–218. 10.1145/2384716.2384777

[B43] Wei C.-H. Allot A. Leaman R. Lu Z. (2019). Pubtator Central: Automated Concept Annotation for Biomedical Full Text Articles. Nucleic Acids Res. 47, W587–W593. 10.1093/nar/gkz389 31114887 PMC6602571

[B44] Wei C.-H. Kao H.-Y. Lu Z. (2015a). Gnormplus: An Integrative Approach for Tagging Genes, Gene Families, and Protein Domains. BioMed Res. Int. 2015, 918710. 10.1155/2015/918710 26380306 PMC4561873

[B45] Wei C.-H. Kao H.-Y. Lu Z. (2012). Sr4gn: A Species Recognition Software Tool for Gene Normalization. PloS one 7, e38460. 10.1371/journal.pone.0038460 22679507 PMC3367953

[B46] Wei C.-H. Leaman R. Lu Z. (2015b). Simconcept: A Hybrid Approach for Simplifying Composite Named Entities in Biomedical Text. IEEE J. Biomed. Health Inform. 19, 1385–1391. 10.1109/jbhi.2015.2422651 25879978 PMC4543296

[B47] Wei C.-H. Phan L. Feltz J. Maiti R. Hefferon T. Lu Z. (2018). Tmvar 2.0: Integrating Genomic Variant Information from Literature with Dbsnp and Clinvar for Precision Medicine. Bioinformatics 34, 80–87. 10.1093/bioinformatics/btx541 28968638 PMC5860583

[B48] [Dataset] Wikipedia contributors (2004a). Disease — Wikipedia, the Free Encyclopedia. Wikimedia Foundation. [Online; accessed 22-July-2021].

[B49] [Dataset] Wikipedia contributors (2004b). Gene — Wikipedia, the Free Encyclopedia. Wikimedia Foundation. [Online; accessed 22-July-2021].

[B50] [Dataset] Wikipedia contributors (2004c). Herbal Medicine — Wikipedia, the Free Encyclopedia. Wikimedia Foundation. [Online; accessed 22-July-2021].

[B51] Wise C. Ioannidis V. N. Calvo M. R. Song X. Price G. Kulkarni N. (2020). Covid-19 Knowledge Graph: Accelerating Information Retrieval and Discovery for Scientific Literature. arXiv preprint arXiv:2007.12731.

[B52] Yu T. Li J. Yu Q. Tian Y. Shun X. Xu L. (2017). Knowledge Graph for Tcm Health Preservation: Design, Construction, and Applications. Artif. Intelligence Med. 77, 48–52. 10.1016/j.artmed.2017.04.001 28545611

[B53] Zheng S. Rao J. Song Y. Zhang J. Xiao X. Fang E. F. (2021). Pharmkg: A Dedicated Knowledge Graph Benchmark for Bomedical Data Mining. Brief Bioinform 22, bbaa344. 10.1093/bib/bbaa344 33341877

[B54] Zheng Z. Liu Y. Zhang Y. Wen C. (2020). “Tcmkg: A Deep Learning Based Traditional Chinese Medicine Knowledge Graph Platform,” in 2020 IEEE International Conference on Knowledge Graph (ICKG), Nanjing, China, August 9–11, 2020 (IEEE), 560–564. 10.1109/icbk50248.2020.00084

[B55] Zhu X. Zhang L. Du J. Xiao Z. (2021). Full-Abstract Biomedical Relation Extraction with Keyword-Attentive Domain Knowledge Infusion. Appl. Sci. 11, 7318. 10.3390/app11167318

[B56] Zhu Y. Che C. Jin B. Zhang N. Su C. Wang F. (2020). Knowledge-Driven Drug Repurposing Using a Comprehensive Drug Knowledge Graph. Health Inform. J 26, 2737–2750. 10.1177/1460458220937101 32674665

